# Wine Barrel Biofilm as a Source of Yeasts with Non-Conventional Properties

**DOI:** 10.3390/microorganisms12050880

**Published:** 2024-04-27

**Authors:** Giorgia Perpetuini, Alessio Pio Rossetti, Arianna Rapagnetta, Giuseppe Arfelli, Roberta Prete, Rosanna Tofalo

**Affiliations:** Department of Bioscience and Technology for Food, Agriculture and Environment, University of Teramo, Via Balzarini 1, 64100 Teramo, Italy; aprossetti@unite.it (A.P.R.); arapagnetta@unite.it (A.R.); garfelli@unite.it (G.A.); rprete@unite.it (R.P.)

**Keywords:** non-*Saccharomyces* yeasts, biofilm, γ-aminobutyric acid, antioxidant activity, oak barrels, vino cotto

## Abstract

This study investigated the main microbial groups characterizing the interior surface of oak barrels from different years (1890, 1895, 1920, 1975, 2008) used in the production of vino cotto. The yeasts were characterized for the following properties: γ-aminobutyric acid (GABA) production, antioxidant activity, air–liquid interfacial biofilm formation, and anthocyanin adsorption capacity. Community-level physiological profile analysis revealed that the microbial communities inside the barrels used the tested carbon sources in different manners. The following yeast species were identified: *Millerozyma farinosa*, *Zygosaccharomyces bisporus*, *Wickerhamiella versatilis*, *Zygosaccharomyces bailii*, *Starmerella lactis-condensi*, and *Zygosaccharomyces rouxii*. All the strains were able to produce GABA, and *S. lactis-condensi*, *Z. bisporus* and *Z. rouxii* were the highest producers (more than 600 mg/L). The *Z. rouxii* and *Z. bailii* strains showed the highest antioxidant activity. Only seven strains out of ten *M. farinosa* formed air–liquid interfacial biofilm. None of the *M. farinosa* strains adsorbed anthocyanins on their cell wall. The other strains adsorbed anthocyanins in a strain-dependent way, and the highest adsorption was observed for the *W. versatilis* strains. The yeasts isolated in this study could be used to increase the functional properties and the quality of fermented foods and beverages.

## 1. Introduction

Fermented beverages have been aged and stored in wooden barrels for centuries. Barrels became prevalent as oil and wine containers about 2000 years ago in Northern Europe, because of the scarcity of clay in those locations [1,2]. Nowadays, wooden barrels are widely used for wine aging, since the flavor compounds extracted from wood, such as phenolic compounds and tannins, can contribute to the definition of the aroma profile of wine [3,4]. Wooden barrels provide an appropriate environment for microorganisms, such as yeasts and bacteria. These microbes can originate from the environment, and they can persist in the barrels even after sanitization procedures [5,6,7]. Microorganisms that live in barrels can be detrimental if they are linked to the creation of unpleasant tastes and the spoiling of the contents [8,9]. These microorganisms can interact with both the barrels themselves and the compounds formed from the wood. The release of wood compounds can serve as a source of nutrients for microbial growth, or it can produce chemicals that have antimicrobial properties [10].

Actually, only a few studies have investigated the microorganisms inhabiting the interior surface of barrels. The majority of them have been performed on barrels used for wine and beer production, or on wooden vats used for cheese making. De Roos et al. [6] examined the microorganisms present on the interior surfaces of different wooden barrels from a traditional lambic beer brewery. *Dekkera*, *Debaryomyces*, *Wickerhamomyces*, *Pediococcus*, *Lactobacillus* and *Acetobacter* were the main genera detected. Guzzon et al. [8] determined the effect of sanitization procedures on the microbial communities present on the interior surface of barrels used for wine production. *Candida* and *Pichia* were the main genera, while *Brettanomyces*/*Dekkera* represented a limited part of the population. A similar approach was used to investigate the microorganisms present in the wooden vats used for cheese making and revealed the presence of several dairy lactic acid bacteria (LAB), including starter LAB (SLAB), responsible for the acidification of curd, and non-starter LAB (NSLAB), implicated in the maturation [11,12,13].

Despite the potential role of barrels as inoculation source of microorganisms—which could influence the characteristics of the final product—no data are available about the microbial communities present in barrels used for the production of traditional products such as vino cotto. Vino cotto (cooked wine) is a sweet wine produced in the Abruzzo and Marche regions. It is obtained according to traditional procedures. The must is heated and concentrated to 30–70% in copper boilers [14]. Once the fermentation ends, vino cotto is aged for at least 5 years in wooden barrels. In a previous study, Battistelli et al. [15] analyzed the cultivable microbiota of mothers of vino cotto collected from barrels of different years (1890, 1895, 1920, 1975, 2008). As stated above, the barrels can act as an additional source of yeasts, which could influence the characteristics of this product. Therefore, this study investigated the microbial groups characterizing the interior surface of the same barrels. Particular attention was paid to yeasts, which were characterized for some non-conventional properties. The strains were tested for the production of γ-aminobutyric acid (GABA) and antioxidant activity, since consumers prefer fermented foods obtained with microorganisms with positive health effects. Moreover, the strains were also tested for air–liquid interfacial biofilm formation, since film-forming yeasts are associated with ethanol reduction [16]. The anthocyanin adsorption capacity was also tested. This activity is associated with wine color stability. 

## 2. Materials and Methods

### 2.1. Sample Origin

The interior surfaces of five 225 L mid-toasted oak barrels of different years (1890, 1895, 1920, 1975, 2008), which were used for the production of vino cotto, were analyzed. Samples were collected in a vino cotto winery located in the Abruzzo region (Italy), in Chieti province. The barrels have never been sanitized, since the must/wine that settles over the years in the bottom of the barrels (mother of vino cotto) is utilized as a sort of inoculum for the production of new batches of vino cotto [15]. Sterile swabs were used for sampling, allowing the swabbing, by rotation, of a fixed area (about 20 cm^2^) on the side panel of the barrels at middle height [6,17]. After swabbing, the sterile swab samples were immediately transferred into sterile plastic tubes containing 20 mL of phosphate-buffered saline (PBS) (137 mM NaCl, 2.7 mM KCl, 10 mM Na_2_HPO_4_, 2 mM KH_2_PO_4_, pH 7.4). The samples were mixed by vortexing for 3 min to resuspend the cells and favor their release from the swab. The swabs were aseptically removed from the PBS solution. The resulting PBS solutions were then analyzed through culture-dependent (plate count) and -independent (community-level physiological profile analysis, CLPP) approaches.

### 2.2. Community-Level Physiological Profile Analysis

Biolog EcoPlates (Biolog Inc., Hayward, CA, USA) were used to evaluate the community-level physiological profile (CLPP) [18]. The microplates contain 31 different carbon-sources, which are closely relevant to the metabolic functions of microbial communities derived from environmental samples, and the control, without carbon source [19]. The samples were centrifuged at 10,000× *g* for 15 min, and the pellet was resuspended in 450 mL of a physiological solution (NaCl 0.85% *w*/*v*). The cell suspension was diluted (1:1000) into the physiological solution, and 150 μL was inoculated into each of the 96 wells of the Biolog EcoPlates according to manufacturer’s instructions. The plates were incubated at 30 °C for 72 h in the OmniLog machine (Biolog Inc.). The utilization of the carbon sources was assessed and measured as cell respiration, as determined by the grade of color (purple) development produced by the NADH reduction of a tetrazolium-based redox dye, included in the wells of the plate with the carbon source [20]. The purple color development was evaluated at 590 nm with a microplate reader (MicroStation; Biolog, Inc.). After completion of the run, the signal data were compiled and exported from the Biolog software and compiled using Microsoft Excel 2021. Three indices were determined, as previously described [11]. The Shannon’s diversity (H) index indicates the substrate utilization pattern. It was calculated as follows: H = −∑pi × ln(pi), where pi is the ratio of the activity of a particular substrate to the sum of the activities of all the substrates. The substrate richness (S) measures the number of different substrates used, and it was calculated as the number of wells with a corrected absorbance > 0.25. The substrate evenness (E) was defined as the equitability of the activities across all the utilized substrates: E = H/Log S [21]. These indices reflected the metabolic functional diversity of the microbial communities, which was similar to the measurements of diversity indices in general ecology [22].

### 2.3. Microbial Count

Ten-fold serial dilutions of the PBS solutions were performed in physiological solution. The cell suspensions were spread-plated and incubated on YPD agar (yeast extract 10 g/L, peptone 20 g/L, dextrose 20 g/L, agar 18 g/L) at 28 °C for 48 h for the yeast enumeration. The LAB were enumerated on the De Man–Rogosa–Sharpe agar (MRS, Oxoid, Milan, Italy) with 100 ppm cycloheximide to inhibit the yeasts’ growth. The plates were incubated at 30 °C for 48 h in microaerophilic conditions. The acetic acid bacteria (AAB) were counted on GYC medium (glucose 100 g/L, yeast extract 10 g/L, calcium carbonate 20 g/L, agar 18 g/L); the plates were incubated at 30 °C for 48 h under aerobic conditions. The cell counts were performed in duplicate. The yeasts were purified by repeated streak-plating on YPD. The purified isolates were stored in YPD broth supplemented with glycerol (20% *v*/*v* final concentration, Sigma Aldrich, Milan, Italy) at −80 °C. The strains belong to the Culture Collection of the Department of BioScience and Technology for Food, Agriculture and Environment (University of Teramo). 

### 2.4. Yeast Identification and Typing

DNA was extracted using the InstaGene^TM^ Matrix according to the manufacturer’s instructions (Bio-Rad, Milan, Italy). The D1/D2 domains of the 26S rRNA gene was amplified for the yeast identification using the NL1 (5′ GCATATCAATAAGCGGAGGAAAAG 3′) and NL4 (5′ GGTCCGTGTTTCAAGACGG 3′) primer pair [23]. The PCR products were purified by ExoSAP-IT (Thermo Fisher, Milan, Italy) according to the manufacturer’s instructions and delivered to BMR Genomics (Padua University, Padua, Italy) for sequencing. The obtained sequences were compared to those available in the GenBank database (http://www.ncbi.nml.nih.gov/BLAST, accessed on 3 January 2024) in order to determine the closest known relative species on the basis of the 26S rRNA gene homology.

The strains were typed by RAPD-PCR with the primer M13 (5′ GAGGGTGGCGGTTCT 3′), as previously described [24]. The Fingerprinting II Informatix^TM^ software program (Bio-Rad, Milan, Italy) was employed for the conversion and normalization of the RAPD-PCR patterns. The similarities among the profiles were calculated by clustering the Pearson’s r correlation matrix using the unweighted pair group method with average (UPGMA) algorithm.

### 2.5. Antioxidant Activity Determination

In order to evaluate the antioxidant activity, the percentage of the reduction in the 1,1 diphenyl-2-picrylhydrazyl (DPPH) radical was calculated according to Gil-Rodríguez et al. [25]. The scavenging activity was assessed by measuring the difference in the absorbance recorded at 517 nm (Jenway 6305 UV/Vis spectrophotometer, Jenway, Essex, UK) between the blank and the sample. The blank contained 0.8 mL of physiological solution, and 1 mL of DPPH. To prepare the samples, 1 mL of yeast culture was harvested by centrifugation (12,000 rpm, 5 min), washed twice with the physiological solution, and the resulting pellet was resuspended in 1 mL of the same solution. The cell suspension (0.8 mL) was transferred into a new tube, where 1 mL of a DPPH solution was added. The antioxidant activity was calculated as follows: Antioxidant activity (%) = [1 − (A517(absorbance of sample)/A517(absorbance of blank))] × 100. The antioxidant capacity was also assessed using the ABTS Antioxidant Assay Kit (Thermo Fisher Scientific, Milan, Italy) according to the manufacturer’s instructions.

### 2.6. γ-Aminobutyric Acid Production

The production of GABA was evaluated according to Yuan et al. [26]. Briefly, the reaction mixture was made up of 0.65 mL of cell supernatant, 0.05 mL of 200 mM sodium borate (pH 9.0), 0.25 mL of 6% phenol (*v*/*v*), and 0.2 mL of 5% (*w*/*v*) sodium hypochlorite. The absorbance was recorded at 630 nm by a Jenway 6305 UV–visible spectrophotometer. A standard solution of GABA (Sigma-Aldrich, Milan, Italy) was used to prepare the standard curve and to calculate the concentration of GABA accumulated in the culture medium.

### 2.7. Air–Liquid Interfacial Biofilm Formation

The formation of an air–liquid interfacial biofilm was evaluated in YPD according to Perpetuini et al. [27]. The strains were grown overnight in YPD broth at 28° C for 48 h. The cells were harvested by centrifugation, and the pellet was resuspended in physiological solution at a final optical density (OD_600nm_) of 1. Then, 10 µL was inoculated in 6-well polystyrene plates and incubated at 28 °C for 10 days. Non-inoculated wells were used as a negative control. Biofilm formation was evaluated daily. All the analyses were performed in triplicate.

### 2.8. Anthocyanin Absorption Capacity

The anthocyanin absorption capacity was evaluated using grape skin yeast peptone dextrose agar, according to Morata et al. [28]. Briefly, YPD plates were supplemented with an anthocyanin solution in a 1:2 ratio. The anthocyanin solution was obtained by macerating red grape skins (*Vitis vinifera* L. cultivar Montepulciano) in a 0.1 M tartaric acid solution for 24 h. The absorption results were observed after the incubation at 30 °C for 48 h. The wine color adsorption (WCA) traits of the strain were evaluated according to Caridi et al. [29], with some modifications. The color analysis of the images was conducted with Adobe Photoshop CS for Windows XP, and red, green, and blue (RGB) were acquired with a 5 × 5 pixel region of interest, and four replicates were considered for each strain. Photoshop’s RGB color mode assigned intensity values ranging from 0 (black) to 255 (white) for each RGB component, enabling differentiation between low WCA (white colony) with higher RGB values and high WCA corresponding to lower RGB values (dark red/brown colony) [29].

### 2.9. Statistical Analysis

The Prism 8.0 program (GraphPad Software Inc., La Jolla, CA, USA) was used to analyze the data and prepare the graphs. The results were expressed as the mean value ± standard deviation. The statistical comparison was performed through a two-way repeated measure ANOVA and a Bonferroni correction was applied. A level of *p* < 0.05 was considered statistically significant. Principal component analysis (PCA) was performed using XLStat 2014 software (Addinsoft, New York, NY, USA).

## 3. Results and Discussion

### 3.1. Community-Level Physiological Profile Analysis

**Figure 1:**
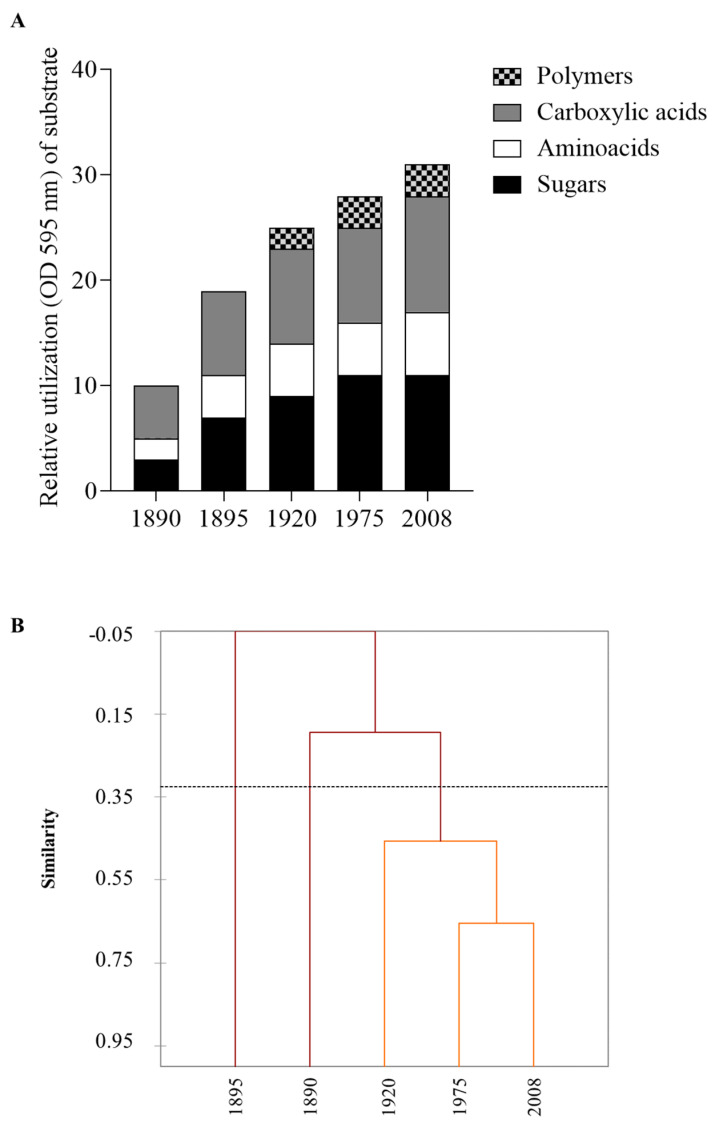
(**A**) Patterns of utilization of the carbon sources by the microbial communities present on the interior surfaces of the barrels. (**B**) Dendrogram showing the similarity of the metabolic profiles of the microbial communities of the different barrels.

The metabolic profiles of the microbial communities inhabiting the interior surface of the oak barrels were assessed by the CLPP technique. This approach could be useful to highlight the differences in the ability to use the carbon sources tested by the microbial communities present in the barrels. The presence/absence of a response in the CLPP may reflect the functional potential and/or a metabolic shift [22]. The obtained data revealed that these communities showed different capacities to utilize the tested carbon sources (Figure 1A). Sugars and carboxylic acids were the main substrates used; polymers were not used by the microorganisms present in the barrels of 1890, 1895, and 1920. In general, the microbial communities associated with the oldest barrels (1890, 1895) used a lower number of substrates than the others (Figure 1A). For instance, only three carbohydrates (glucose 1-phosphate, D-L α-glycerol phosphate, and β-methyl D-glucoside) were metabolized by microbes present in the barrel of 1890. The metabolic potential was also estimated by determining three indices (S, H, and E), which showed differences based on the ability, or lack of ability, to use the carbon sources. The metabolic function was increased in the barrels of 1975 and 2008; they showed higher values of the H and S indices than the others (Table 1). The E index, providing a measure of the statistical significance (equitability) of the values of the H and S indices, confirmed the significant (*p* < 0.05) differences between the samples. Even if this technique could not provide information on the microbial taxonomic structure and its composition, the results evidenced that the microbial metabolic activities were affected by the barrel age. The dendrogram obtained allowed us to visualize the relationship between the samples (Figure 1B). It showed that the microbial communities isolated from the most recent barrels (1920, 1975, 2008) had a similar metabolic potential, while those isolated from the 1890 and 1895 barrels were different, from a metabolic point of view, not only compared to the previous ones but also among themselves. The fact that the microbial communities of the newer barrels (1975, 2008) used more carbon sources than the others suggested that the microorganisms in the older barrels have changed their metabolism by blocking some metabolic activities in favor of others, which are more useful to survive in this niche. Probably, the species and strains have been selected over time thanks to their metabolic plasticity. The concept of the loss of less-adapted species or strains is known as competitive exclusion. Through the selection of traits that reduce dependence on a common resource, populations may shift toward coexistence. This is known as niche partitioning, whereby competition is avoided through the utilization of different resources [30].

### 3.2. Microbial Count

**Figure 2:**
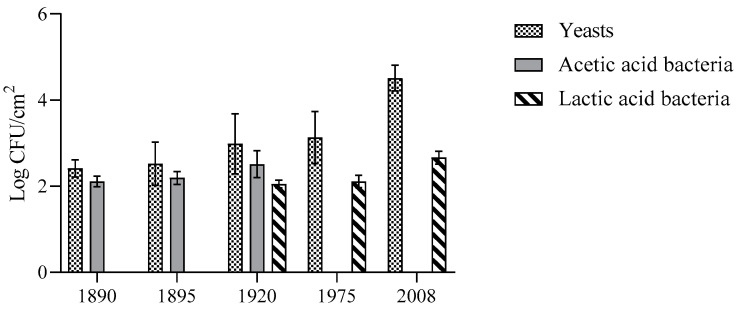
Viable counts of yeasts, acetic acid bacteria and lactic acid bacteria in the different barrels.

The cultivable microorganisms inhabiting the barrels were evaluated by plate count. Yeasts were the main microorganisms detected, with values ranging from 2.4 Log CFU/cm^2^ to 4.5 Log CFU/cm^2^. AAB were only detected in barrels from 1890, 1895, and 1920, with values of 2.11 Log CFU/cm^2^, 2.19 Log CFU/cm^2^, and 2.51 Log CFU/cm^2^, respectively. LAB were only present in more recent barrels (1975 and 2008), with values higher than 2 Log CFU/cm^2^ (Figure 2). Similar data were obtained by Battistelli et al. [15], who studied the microorganisms present in the mothers of vino cotto. Also in this case, yeasts and LAB were more present in more recent barrels (1975 and 2008); even if they showed higher values of viable counts than those of the corresponding barrels. The extractable compounds from wood barrels are finite, and the rate of extraction and the amounts of compounds extracted diminish as the barrel is used in successive years [31]. Probably, more recent barrels are richer in compounds able to support the growth of LAB, which are generally found in nutrient-rich habitats [32].

AAB were detected in the barrels of 1890, 1895, and 1920, with values of about 2 Log CFU/cm^2^. The occurrence of AAB in these barrels is probably due to the oxygen availability, which is higher in the oldest barrels due to their increased porosity. In general, the microbial counts of yeasts, LAB, and AAB were lower than those found in the respective mothers, as expected. In fact, the wood surface represents a more stressful environment in terms of nutrients, oxygen availability, water activity, etc. Moreover, the microorganisms could be in a viable, not cultivable state or engrained deeper in the wood; therefore, they cannot be sampled with swabs. Similar data were obtained by De Roos et al. [6], who analyzed the interior part of barrels used for beer production. In this case, the yeasts were also the predominant microbial group, and similar values to those reported in this study were obtained. Guzzon et al. [8] observed an increase in yeast density according to the age of wine barrels. Barriques, which had been used for more than 3 years, showed a yeast concentration below 3 Log CFU/cm^2^, 1 Log unit higher than that found in more recent vats. However, it should be considered that the barrels used in that study were subjected to regular cleaning treatments; on the contrary, the barrels analyzed in this study have never been sanitized. The differences detected among studies in terms of the microbial counts are not surprising since they depend on several factors, including the type and age of the barrels and the sanitization treatments. Moreover, even barrels of the same type and age can harbor a heterogeneous microbial composition [6].

**Figure 3:**
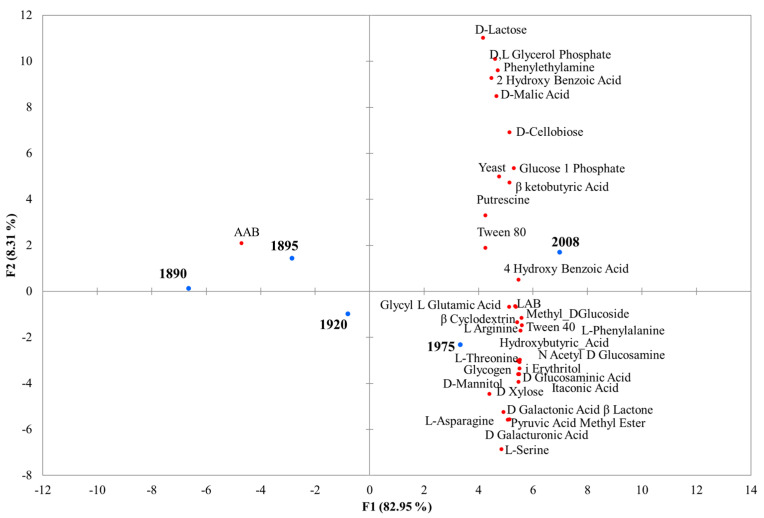
The biplot (score and loading) of the first two principal components, which showed 91.26% of the cumulative variance.

PCA analysis was performed to discriminate the samples in terms of the carbon usage efficiency and microbial counts (Figure 3). The differences were directly reflected by the position of the points in the principal vector space. The samples closest to one another and far from the plot origin were positively correlated, while the variables opposite one another on the plot were negatively correlated. The chemical compounds that contribute most to the variance were L-phenylalanine, β-cyclodextrin, N-acetyl D-glucosamine, D-glucosaminic acid, glycyl L-glutamic acid, and i-erythritol. The barrels of 1890 and 1895 were grouped together and were characterized by the occurrence of AAB. The barrels from 2008 clustered alone and were characterized by D-lactose, D-L glycerol phosphate, phenylethylamine, 2 hydroxy benzoic acid, D-malic acid, D-cellobiose, glucose 1-phosphate, β-ketobutyric acid, putrescine, Tween 80, 4 hydroxy benzoic acid, and yeasts. Finally, the barrels from 1975 were differentiated from the others for the other compounds and LAB.

### 3.3. Strain Identification and Typing

The yeasts were identified by sequencing the D1/D2 domain of the 26S rRNA gene [23]. All the sequences obtained displayed similarity values ranging from 99 to 100% (Supplementary Table S1). The species detected were *Zygosaccharomyces bisporus* (8 isolates), *Zygosaccharomyces bailii* (3 isolates), *Meillerozyma farinosa* (10 isolates), *Starmerella lactis-condensi* (2 isolates), *Wickeramiella versatilis* (2 isolates), and *Zygosaccharomyces rouxii* (1 isolate). The barrels of 1890 and 1895 were characterized by the occurrence of only two species, *M. farinosa* and *Z. bisporus*. These two species were also detected in the barrels of 1920, together with *W. versatilis*, *S. lactis-condensi*, and *Z. rouxii*. In the 1975 and 2008 barrels, yeasts belonging to the following species were isolated: *S. lactis-condensi*, *Z. bailii*, *W. versatilis*, *Z. bisporus*, and *M. farinosa*, *Z. bisporus*; and *Z. bailii*, respectively. The species detected are osmophilic yeasts, and only *S. lactis-condensi* has been detected in the mothers of vino cotto and throughout the fermentation process of vino cotto [16,24]. *Millerozyma farinosa* is a halotolerant diploid yeast belonging to the Saccharomycetaceae family, which produces high yields of glycerol and xylitol. This yeast is able to release a salt-mediated killer toxin that kills competitive strains [33]. It is commonly found in foods such as fermented alcoholic beverages, soybean paste, and miso [34]. *Wickerhamiella versatilis* is closely related to the genus *Starmerella* [35]. It is an osmophilic species; in fact, it has been isolated in honey and soy sauce [36,37,38]. *Z. bailii* has always attracted the interest of researchers for its ability to survive in stressful environments such as wine, vino cotto, mustard, fruit juices, and dairy products [24,39]. In the wine sector, its use in mixed starters with *Saccharomyces cerevisiae* has been proposed to improve the production of ethyl esters. Furthermore, it is successfully used for the production of balsamic vinegar [40]. *Z. rouxii* shows osmophilic and fructophilic characteristics. It plays a key role in defining the aromatic profile of balsamic vinegar [41]. *Z. bisporus* is a fructophilic yeast isolated from various foods, such as honeydew, grapes affected by sour rot, and mezcal [42]. The differences obtained in terms of species detected, suggested that the age of the barrels could influence the viable microorganisms present on their inner surfaces. This result is in agreement with the CLPP analysis, which revealed the different ability of the microbial communities present in the barrels to use the tested carbon sources. The reduced biodiversity detected in terms of the microbial species could be related to the selective pressure exerted by the environmental conditions characterizing this niche, such as nutrient starvation, which selected only some dominant species.

**Figure 4:**
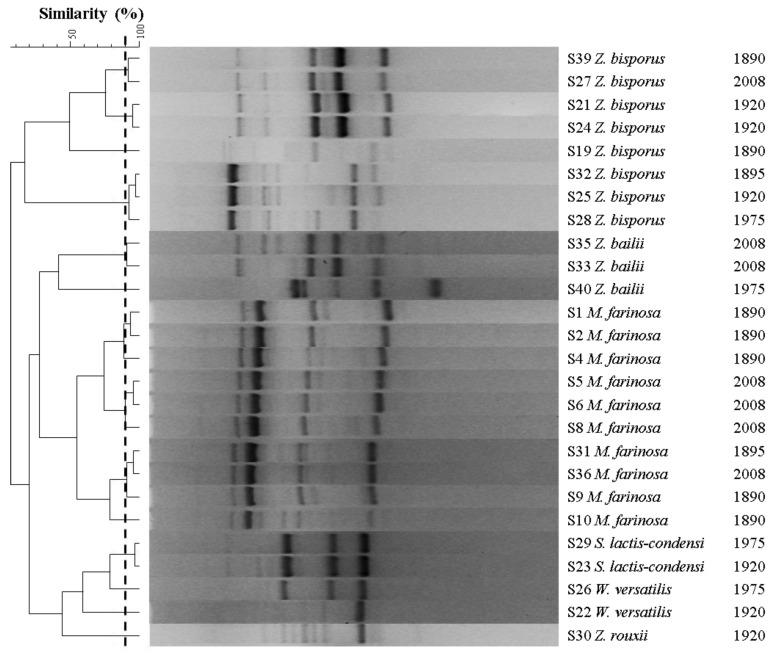
RAPD-PCR cluster analysis. Unweighted pair group method with arithmetic mean (UPGMA) dendrogram derived from comparison of the RAPD-PCR patterns of the yeast isolates obtained with the primer M13.

RAPD-PCR analysis was performed in order to assess the biodiversity among the isolates and deduplicate the collection [43]. Banding patterns with a level of similarity higher than 90% were considered as a biotype. The UPGMA dendrogram is shown in Figure 4. RAPD-PCR resulted in a coherent classification at the species level. Four biotypes were detected for *Z. bisporus* and two for *Z. bailii*. The 10 *M. farinosa* strains were grouped into 4 biotypes. The two isolates of *S. lactis-condensi* clustered together, while the two *W. versatilis* strains belonged to two different clusters. This low RAPD-PCR diversity can be explained by the supposed prevalence of a small number of dominant species, or “core” strains, selected by the stressful conditions imposed by this peculiar environment. Some biotypes are present in different years, suggesting that the selective pressure exerted by the environmental conditions, selected well-adapted biotypes that are able to survive over the years [44].

### 3.4. Production of γ-Aminobutyric Acid

γ-aminobutyric acid is a neurotransmitter in the central nervous system with hypotensive, tranquilizing, and diuretic effects [45]. The GABA-shunt pathway consists of three enzymes: (i) glutamate decarboxylase, which catalyzes the conversion of glutamate to GABA; (ii) GABA aminotransferase, involved in the conversion of GABA and α-ketoglutarate to succinate semialdehyde and glutamate; and (iii) succinate semialdehyde dehydrogenase, catalyzing the NAD(P)-dependent conversion of succinate semialdehyde into succinate [45]. Strains sharing the same RAPD profile produced a similar amount of GABA, independently of their origin. A certain variability was observed at the strain level. In fact, the *M. farinosa* strains produced an amount of GABA ranging from 274.98 mg/L (S9) to 296.71 mg/L (S4); the *Z. bisporus* strains from 540.51 mg/L (S39) to 616.39 mg/L (S28); the *Z. bailii* strains from 535. 22 mg/L (S40) to 546.55 mg/L (S36). *W. versatilis* S26 and S22 released a quantity of GABA of 417.68 mg/L and 443.96 mg/L, respectively, and *S. lactis-condensi* S23 and S29 produced about 630 mg/L of GABA (Table 2). To the best of our knowledge, this is the first time that the production of GABA was demonstrated in these yeast species. However, it has been described in other yeast species, including *Saccharomyces cerevisiae*, *Rhodotorula mucillaginosa*, *Debaryomuyces hansenii*, *Kazachstania unispora*, *Metschnikowia reukaufii*, *Pichia guilliermondii*, *Pichia scolyti*, *Pichia silvicola*, *Sporobolomyces carnicolor*, *Sporobolomyces ruberrimus*, and *Kluyveromyces marxianus* [46,47,48]. It should be noted that the strains isolated in this study produced higher amounts of GABA than those reported in other studies [46,47]. The higher values found could represent a mechanism of adaptation of the yeasts to the stressful growth conditions to which they are subjected (e.g., osmotic stress, nutrient deficiency, oxygen availability, presence of antimicrobial compounds extracted from wood, etc.). In fact, the production of GABA seems to play an important role in stress resistance [49]. Its synthesis consumes protons to form H_2_O and enhances the acid-stress response [50]. Moreover, according to Cao et al. [51], this compound is important in the tolerance of oxidative stress. The GABA shunt plays a key role in the resistance of *S. cerevisiae* to some antimicrobial compounds, such as furfural, acetic acid, and phenolic compounds [49]. Ji et al. [52] demonstrated that the intra-cellular accumulation of GABA in *Candida glycerinogenes* improves its tolerance to osmotic stress, suggesting that it could act as a compatible solute. The strains isolated in this study could be used as starter cultures to obtain GABA-fortified foods. In fact, even if GABA is present in several foods (e.g., vegetables, grains, milk), its content is quite low. Microbial fermentation represents a good strategy to increase the GABA content in some fermented foods (e.g., dairy products) [53]. Therefore, the selection of new high-GABA-producing strains from ecological niches, different from foods, should remain a focus of interest in future research because it can offer concrete opportunities for the design of new functional foods.

### 3.5. Antioxidant Activity of Strains

The strains were tested for their antioxidant activity, measuring the DPPH and the ABTS free radical-scavenging activities. The results are reported in Table 3. The *Z. bailii*, *S. lactis-condensi*, and *Z. rouxii* strains showed the highest DPPH and ABTS radical-scavenging activity. The lowest activity was shown by the *W. versatilis* strains. A certain intra-species and inter-species variability was detected, and similar results were also detected by other authors [25,54]. It should be noted that, strains clustering together with the RAPD-PCR analysis, showed similar values independently of the origin, as previously observed for GABA production. According to Gil-Rodríguez et al. [25], yeasts can be divided into five groups according to the DPPH radical-scavenging activity: very low (<20%), low (20–30%), good (30–40%), very good (40–50%), and excellent (>50%). Based on this grouping, the strains tested in this study can be classified as follows: S9 (*M. farinosa*), S19 (*Z. bisporus*), S26, and S22 (*W. versatilis*) showed very low activity; S1, S2, S4, S31, S36 (*M. farinosa*), S21, S24, and S28 (*Z. bisporus)* showed low activity; S5, S6, S8, S10 (*M. farinosa*), S39, and S27 (*Z. bisporus)* showed good activity; S32, S25 (*Z. bisporus*), S33, S35 (*Z. bailii*), S23, S29 (*S. lactis-condensi)* showed very good activity; and S40 (*Z. bailii*) and S30 (*Z. rouxii*) showed excellent activity. Similar results were obtained with the ABTS method. In this case, the radical-scavenging activity ranged from about 33% (S22, S26) to 89.8% (S30). The strong antioxidant activity of *Z. rouxii* has already been reported by Naylin et al. [55], who proposed its use as a source of natural antioxidants. The antioxidant activity of *M. farinosa* could be due to its ability to produce glutathione [56]. The antioxidant activity of yeasts mainly relies on the content of (1,3), (1,6) β-D-glucan and other β-glucans found in the cell wall, and of some antioxidant enzymes like superoxide dismutase, glutathione peroxidase, and catalase [40,57]. Moreover, glucans from different sources and with different molecular weights show different antioxidant activities [58]. This last piece of evidence suggested that the intra- and inter-species differences detected could be linked to intra- and inter-specific differences in the composition of the cell wall [59]. It can also be hypothesized that the selective pressure exerted by this peculiar ecological niche has induced a remodeling of the cell wall to better cope with stress. It has been estimated that approximately 1200 genes display a cell-wall-related phenotype [60]. It is therefore not surprising that the cell wall can vary in terms of composition and thickness depending on the environmental conditions [61], influencing the antioxidant activity of yeasts.

The strains showing very good and excellent activity should be proposed as healthful microorganisms for the production of functional foods or natural antioxidant supplements in the food industry.

### 3.6. Air–Liquid Interfacial Biofilm Formation

**Figure 5:**
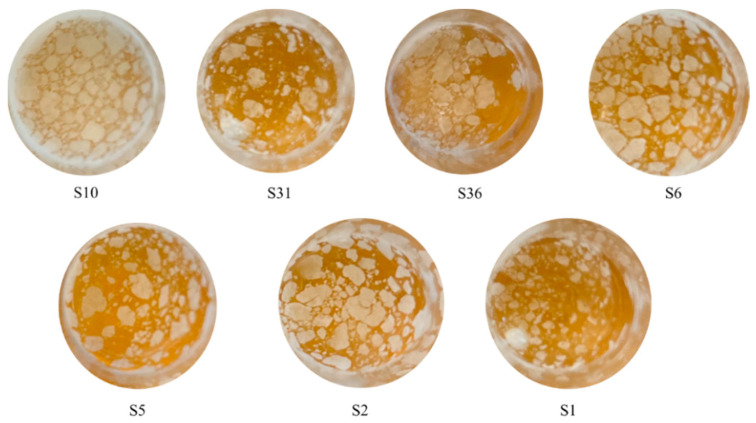
Air–liquid interfacial biofilm formation by the tested strains in YPD.

The strains were also tested for their ability to form an air–liquid interfacial biofilm or velum (Figure 5). The formation of a velum is an adaptive mechanism that is activated in yeasts in response to nutrient starvation. The formation of the velum has been extensively studied in *S. cerevisiae* flor strains. In these strains, ammonium depletion is the key factor in the induction of air–liquid biofilm formation, and *FLO*11 gene expression, which allows the yeast cells to rise to the liquid surface [62].

The wine–air interphase is characterized by a high dissolved oxygen content and constitutes the place where the velum is developed. Therefore, flor yeast metabolism is purely oxidative. They use ethanol and glycerol as the major carbon sources in absence of fermentable sugars [63]. 

The obtained data revealed that only 7 strains (out of 10) of *M. farinosa* produced the velum. The strains were not able to produce a continuous film, with only the exception of the S1 strain. The other strains produced only small islands of aggregated cells. The ability of the *M. farinosa* strains to form the velum is due to the aerobic metabolism. Moreover, this species is phylogenetically related to the *Candida* genus, which is associated with the velum formation [64]. The determination of this activity is important, since film-forming yeasts are considered spoilage microorganisms during wine fermentation since they form floating “flowers” with a quick progression to a thick, dusty pellicle [65]. However, their role in winemaking has been re-evaluated since their use in combination with *S. cerevisiae* resulted in an improvement in the wine aroma, the degradation of malic acid, and the reduction of the ethanol content [16]. This last ability could be of interest since climate change has induced several modifications to the grape composition, and the increase in the sugar content is one of them.

### 3.7. Anthocyanin Adsorption Capacity

The strains were tested for their ability to adsorb anthocyanins. The utilization of anthocyanin-enriched agar plates allowed to identify the strains that can adsorb greater amounts of anthocyanins on their cell walls, with a negative impact on the color of wines [28,29]. All the strains absorbed anthocyanins, with the only exception of *M. farinosa* strains; in fact, the colonies were white (Supplementary Figure S1). In order to better identify the differences among the species and strains, the RGB component was analyzed (Table 4). In agreement with the color of the colonies, the *M. farinosa* strains showed the highest RGB values, confirming the inability of these strains to adsorb anthocyanins. The lowest values were obtained for *W. versatilis*. The adsorption capacity seemed to be strain-dependent. This variation could be due to differences in the composition and structure of the cell walls, as previously suggested [28]. The inability to adsorb anthocyanins is an attractive trait for preserving the color of red wine [28,29]. The selection of wine starters characterized by different anthocyanin-adsorbing activity can have the following effects on wine quality: the protection of red wines; the removal of residual color when making white wine; and the protection of phenolic compounds responsible for the antioxidant activity of wine.

## 4. Conclusions

The exploration of the microbial biodiversity of natural ecosystems is a promising and challenging approach in the quest for novel potential starters for use in the production of fermented foods and beverages.

In this study, the application of CLPP analysis revealed that the environment exerts a certain pressure on microbial metabolism. In fact, the microbial communities inhabiting barrels of different ages used the tested carbon sources in different ways, suggesting differences in their technological and physiological properties. Therefore, the yeasts were isolated and characterized in order to select novel non-conventional yeast strains. The use of non-conventional yeasts provides interesting opportunities for artisans to diversify their product palette or to create innovative, high-quality fermented foodstuffs. The strains isolated in this study could be useful for improving the production of fermented beverages and foods, improving their quality (e.g., color) and healthy properties, increasing the content of GABA and improving the antioxidant activity. However, further studies are necessary to test these strains in fermentation trials and to ensure biosafety in order to propose their use as starter cultures.

## Figures and Tables

**Table 1:** Average values of the diversity (H), richness (S), and evenness (E) of the different samples.

Index	Sample					*p* Value
	1890	1895	1920	1975	2008	
S	10 ± 1.12	19 ± 0.98	25 ± 0.55	28 ± 1.21	31 ± 1.01	0.000470
E	1.38 ± 0.21	1.55 ± 0.45	1.78 ± 0.09	2.37 ± 0.16	2.55 ± 0.11	0.010795
Shannon	2.73 ± 0.12	2.95 ± 0.04	3.05 ± 0.17	3.17 ± 0.76	3.38 ± 0.08	0.000358

**Table 2:** GABA produced by the tested strains.

Strain	Species	mg/L
S1	*M. farinosa*	292.06 ± 78.4
S2		291.05 ± 83.8
S4		296.71 ± 56.7
S5		232.92 ± 57.1
S6		234.66 ± 61.7
S8		239.11 ± 59.4
S31		277.73 ± 74.1
S36		278.75 ± 83.3
S9		274.98 ± 59.2
S10		267.17 ± 71.9
S39	*Z. bisporus*	540.54 ± 23.4
S27		541.01 ± 65.8
S21		586.46 ± 66.7
S24		585.87 ± 41.7
S19		512.25 ± 23.5
S32		613.96 ± 89.6
S25		614.01 ± 99.7
S28		616.39 ± 51.2
S26	*W. versatilis*	417.68 ± 27.3
S22		443.96 ± 95.3
S33	*Z. bailii*	546.05 ± 20.8
S35		546.55 ± 53.7
S40		535.22 ± 77.8
S23	*S. lactis-condensi*	631.22 ± 35.7
S29		630.54 ± 43.9
S30	*Z. rouxii*	580.03 ± 51.5

**Table 3:** Antioxidant activity of the tested strains.

Strain	Species	Radical Scavenging (%)	
		DPPH	ABTS
S1	*M. farinosa*	29.6 ± 2.4	65.2 ± 12.8
S2		29.3 ± 4.9	64.8 ± 9.8
S4		31.5 ± 3.8	63.9 ± 15.9
S5		38.6 ± 5.9	73.8 ± 14.8
S6		38.4 ± 3.8	72.9 ± 17.6
S8		39.2 ± 6.4	74.6 ± 9.8
S31		22.8 ± 5.8	61.5 ± 17.8
S36		23.6 ± 6.9	62.2 ± 8.6
S9		24.6 ± 3.9	60.7 ± 12.5
S10		36.8 ± 3.7	70.9 ± 13.9
S39	*Z. bisporus*	36.7 ± 7.9	56.8 ± 14.9
S27		36.1 ± 8.4	55.9 ± 19.9
S21		21.6 ± 2.4	50.6 ± 6.5
S24		20.8 ± 3.5	50.2 ± 12.6
S19		18.7 ± 3.5	30.4 ± 13.7
S32		44.2 ± 5.9	61.9 ± 14.6
S25		44.5 ± 5.2	62.2 ± 9.8
S28		46.8 ± 7.5	58.8 ± 12.3
S26	*W. versatilis*	19.8 ± 2.5	33.8 ± 14.9
S22		17.3 ± 1.33	33.7 ± 15.4
S33	*Z. bailii*	42.6 ± 12.5	82.9 ± 13.7
S35		43.3 ± 7.2	81.7 ± 9.4
S40		51.3 ± 6.9	85.6 ± 21.6
S23	*S. lactis-condensi*	36.9 ± 5.9	66.6 ± 19.7
S29		36.6 ± 7.8	68.8 ± 22.6
S30	*Z. rouxii*	62.6 ± 6.4	89.8 ± 13.6

**Table 4:** Red, green and blue components of the tested strains on grape skin YPD medium.

Strain	Species	R *	G **	B ***
S1	*M.* *farinosa*	210.5 ± 2.56	168 ± 6.12	133.75 ± 11.65
S2		215.25 ± 8.12	171 ± 5.47	135.5 ± 13.28
S4		217 ± 3.15	171.5 ± 12.15	137.5 ± 12.44
S5		214.75 ± 12.65	186.75 ± 6.15	153.5 ± 9.87
S6		210.5 ± 5.56	181 ± 15.31	156.5 ± 14.55
S8		208.5 ± 3.95	180.75 ± 9.32	148 ± 13.67
S31		223.25 ± 10.23	188.5 ± 4.14	141.5 ± 9.32
S36		222.25 ± 6.77	185 ± 12.63	141 ± 11.45
S9		221.88 ± 12.45	187 ± 8.11	142.75 ± 15.36
S10		193 ± 13.65	167.25 ± 6.32	132.75 ± 11.47
S39	*Z. bisporus*	173.75 ± 19.12	100.75 ± 7.67	53.75 ± 8.23
S27		179.5 ± 5.47	101.5 ± 5.14	55 ± 4.57
S21		122.25 ± 6.45	58 ± 3.24	42.25 ± 7.21
S24		123.75 ± 5.12	61 ± 8.71	41.5 ± 6.29
S19		100.75 ± 3.44	57.75 ± 9.15	54.75 ± 4.58
S25		125.5 ± 11.36	85.75 ± 6.78	61.75 ± 9.14
S32		125.25 ± 9.55	87.25 ± 9.15	57.75 ± 3.555
S28		127 ± 4.65	88.25 ± 6.33	58 ± 7.35
S26	*W. versatilis*	97.75 ± 3.66	59.5 ± 8.15	48.5 ± 2.69
S22		99 ± 2.47	61.5 ± 6.31	46 ± 8.12
S33	*Z. bailii*	120.25 ± 9.78	59.25 ± 7.11	46.75 ± 4.53
S35		119.5 ± 7.62	59.5 ± 8.36	45 ± 5.78
S40		124.5 ± 5.99	76 ± 11.52	53.75 ± 6.99
S29	*S. lactis-condensi*	99.5 ± 4.33	61 ± 9.74	44.5 ± 7.96
S23		101.25 ± 7.89	63 ± 4.79	43.25 ± 6.32
S30	*Z. rouxii*	104.25 ± 12.65	57.5 ± 9.54	44.25 ± 6.12

* R: red component; ** G: green component, *** B: blue component.

## Data Availability

Data are contained within the article.

## Supplementary Materials

The following supporting information can be downloaded at https://www.mdpi.com/article/10.3390/microorganisms12050880/s1, Figure S1: Anthocyanin adsorption by the yeast cell walls; Table S1: Yeast species detected in the different samples.
