# Supplementary material for: Wine Barrel Biofilm as a Source of Yeasts with Non-Conventional Properties
Source: Microorganisms. 2024 Apr 27;12(5):880. doi: 10.3390/microorganisms12050880 (PMC11123285; doi:10.3390/microorganisms12050880)
Supplement: Supplementary file 1 [file microorganisms-12-00880-s001.zip › microorganisms-2946418-supplementary.pdf]

Supplementary Table S1. Yeast species detected in the different samples

Strain	Species	Origin	Accession number	Similarity (%)
**S1**	*M. farinosa*	1890	FN565484	100
**S10**				
**S2**				
**S9**				
**S4**				
**S19**	*Z. bisporus*		AF399790	
**S39**				
**S31**	*M. farinosa*	1895	FN565484	
**S32**	*Z. bisporus*		AF399790	
**S21**	*Z. bisporus*	1920	NG058447	
**S24**			EF460531	
**S25**			AY046115	
**S22**	*W. versatilis*		KY106880	
**S23**	*S. lactis-condensi*		KY106546	
**S30**	*Z. rouxii*		KY110285	
**S29**	*S. lactis-condensi*	1975	KY106546	
**S40**	*Z. bailii*		KY110241	
**S26**	*W. versatilis*		KY106880	
**S28**	*Z. bisporus*		AF399790	
**S27**	*Z. bisporus*	2008	AY046115	
**S33**	*Z. bailii*		KY110241	
**S35**	*Z. bailii*			
**S36**	*M. farinosa*		FN565484	
**S6**				
**S8**				
**S5**				

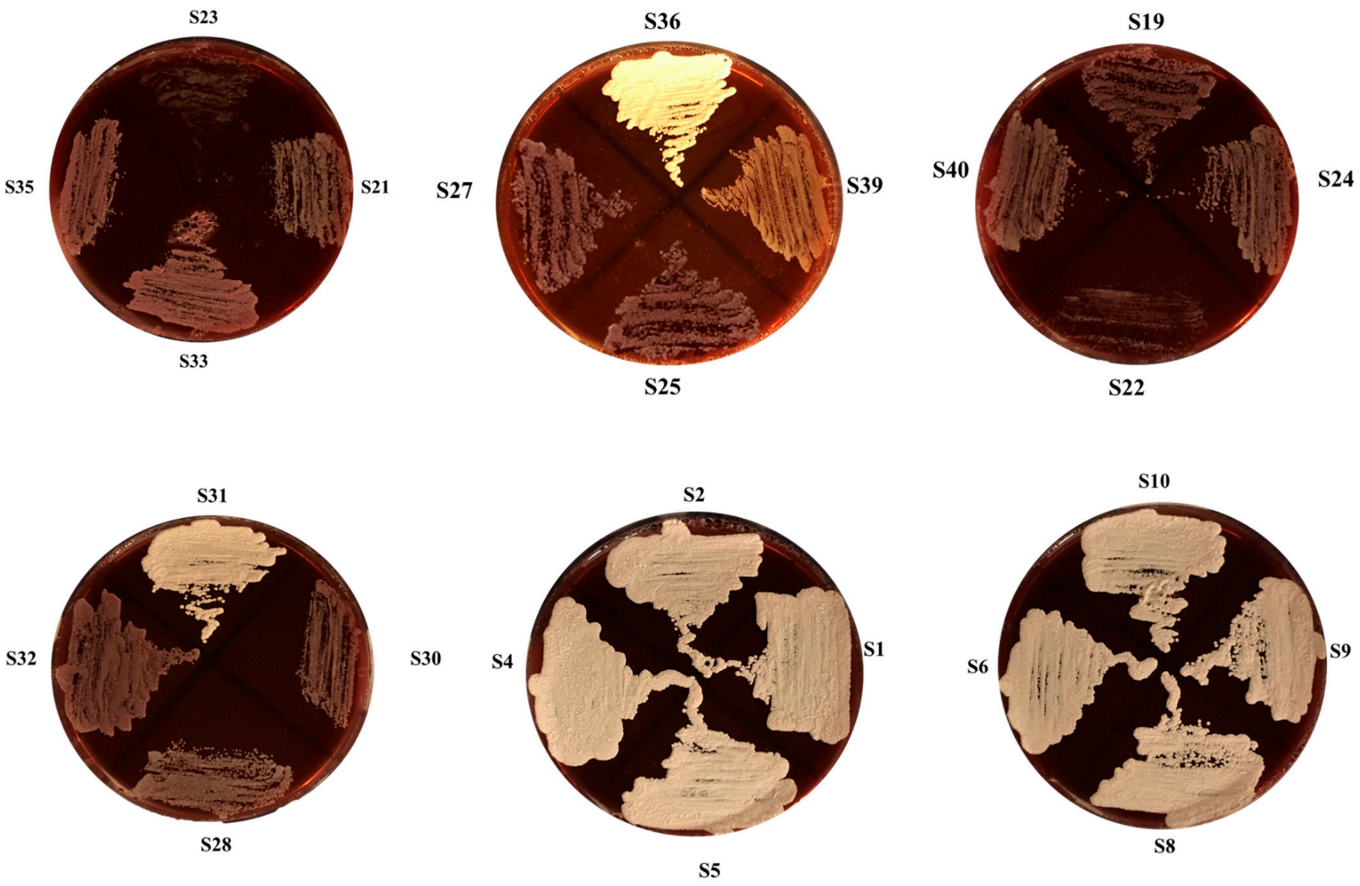

Supplementary Figure S1. Anthocyanins adsorption by yeast cell walls
